# Clonal Population of *Mycobacterium tuberculosis* Strains Reside within Multiple Lung Cavities

**DOI:** 10.1371/journal.pone.0024770

**Published:** 2011-09-14

**Authors:** Viral Vadwai, Gustad Daver, Zarir Udwadia, Meeta Sadani, Anjali Shetty, Camilla Rodrigues

**Affiliations:** P. D. Hinduja National Hospital and Medical Research Centre, Mahim, Mumbai, India; Fundació Institut Germans Trias i Pujol, Universitat Autònoma de Barcelona CibeRES, Spain

## Abstract

**Background:**

Unsuccessful treatment outcomes among patients with multi-/extensively- drug resistant tuberculosis (TB) have hampered efforts involved in eradicating this disease. In order to better understand the etiology of this disease, we aimed to determine whether single or multiple strains of *Mycobacterium tuberculosis* (MTB) are localized within lung cavities of patients suffering from chronic progressive TB.

**Methodology/Findings:**

Multiple cavity isolates from lung of 5 patients who had undergone pulmonary resection surgery were analyzed on the basis of their drug susceptibility profile, and genotyped by spoligotyping and 24-loci MIRU-VNTR. The patients past history including treatment was studied. Three of the 5 patients had extensive drug resistant TB. Heteroresistance was also reported within different cavity isolates of the lung. Both genotyping methods reported the presence of clonal population of MTB strain within different cavities of the each patient, even those reporting heteroresistance. Four of the 5 patients were infected with a population of the Beijing genotype. Post-surgery they were prescribed a drug regimen consisting of cycloserine, a fluoroquinolone and an injectable drug. A 6 month post-surgery follow-up reported only 2 patients with positive clinical outcome, showing sputum conversion.

**Conclusion:**

Identical spoligotype patterns and MIRU-VNTR profiles between multiple cavities of each patient, characterize the presence of clonal population of MTB strains (and absence of multiple MTB infection).

## Introduction

Infection due to a single strain of *Mycobacterium tuberculosis* (MTB) was considered as the cause of active tuberculosis (TB) until early 1970's when phage typing reported the presence of more than one infecting strain in a single patient [1], [2]. Since then active TB is thought as a result of either due to: primary infection with a single strain of MTB (primary TB)/after endogenous reactivation of primary infection i.e. with same strain (relapse)/exogenous infection with a second MTB strain (reinfection) or due to simultaneous infection with two or more strains of MTB (mixed infection) [1], [3]. The latter two forms of TB, possibly the most vulnerable due to the phenomenon of heteroresistance has been proven in clinical tuberculosis [4]. Infection with different MTB strains each having different drug susceptibility pattern makes it difficult to effectively treat the patient with a correct combination of anti-tubercular drugs, leading to multi-/extensive- drug resistant (M/XDR) cases. In such cases, the tubercle bacilli overcomes the host immune defense system and does not respond to the anti-tubercular treatment (ATT) leading to chronic progressive disease with formation of cavities, fibrotic lesions and tissue necrosis in the lungs of patients. Previous studies have shown that pulmonary resection has shown to be successful in treatment of such drug resistant cases [5], [6]. Thus for effective pre- and post-operative anti-tubercular treatment, accurate identification and differentiation between MTB strains is of prime importance. Few studies have shown the utility of 24-loci Mycobacterial Interspersed Repetitive Units-Variable Number Tandem Repeats (MIRU-VNTR), a fingerprinting tool in detection of mixed infection and sub-clonal population [7], [8].

To determine the presence/absence of multiple MTB strains within lung cavities of patients suffering from chronic progressive TB, we analyzed multiple cavities from lungs of each of the 5 patients who underwent pulmonary resection surgery by determining the drug susceptibility profile of each cavity isolate and further characterizing the bacterial populations present by both, spoligotyping and 24-loci MIRU-VNTR.

## Results

### Patient: clinical findings, treatment history and hospitalization characteristics

**Figure 1:**
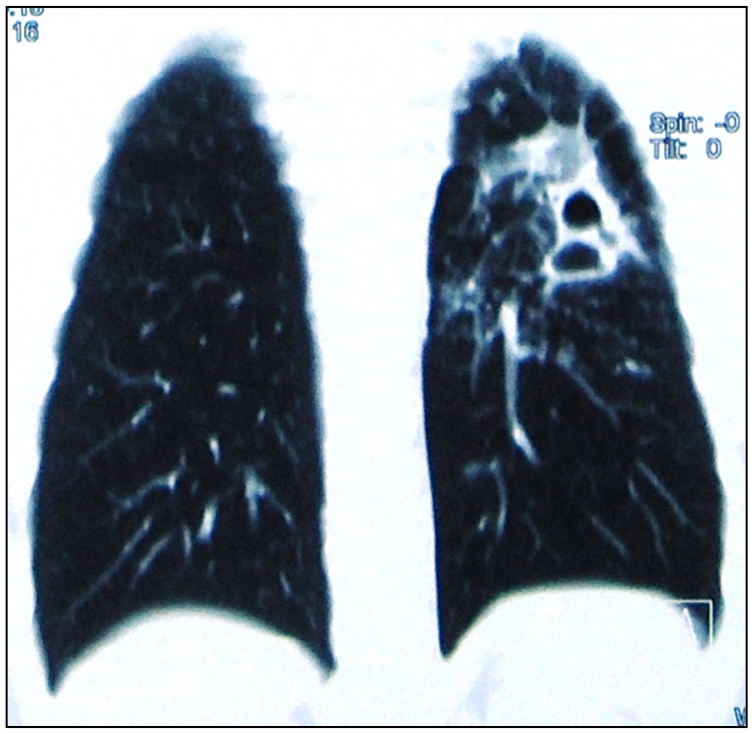
Radiological image of the lungs of a patient before surgery.

**Figure 2:**
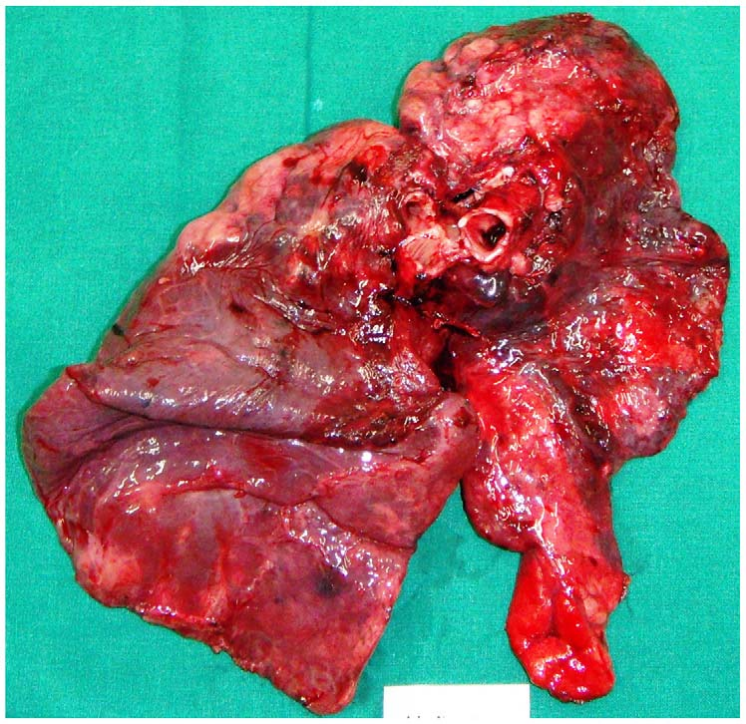
Resected lung with multiple cavities, area of tissue necrosis and granulomatous inflammation.

Three patients (patients 1, 3 and 5) had previous episodes of TB for which they were treated successfully to complete recovery. None of the patients were HIV seropositive (Table 1. provides details about the demographic and clinical characteristics of patients). All patients had unilateral lung disease with complete destruction, reduced air entry on the affected side and were on anti-tubercular treatment for at least 36 months prior to the date of surgery. Each patient had at least received an average of 8±0.83 (mean number of anti-tubercular drugs ± Standard Deviation) anti-tubercular drugs. Table 2. represents a detailed treatment history of patients pre- and post- pulmonary resection surgery. Histological analysis of all cavities confirmed the presence of TB: caseation necrosis with epitheloid cells, visual presence of acid fast bacilli and formation of granulomas. Radiologal scans of all patients showed the presence of more than one fibrous cavitory lesions in the affected lung. Fig. 1. shows the radiological scan of patients lung prior to surgery and Fig. 2. shows the resected lung. Patient 1, 2, 3 underwent surgery due to persistent smear positive status; patient 4, to avoid complications due to hemoptysis and patient 5, a result of continuous discharge of pus from the right lung and expired within 72 hours post-surgery due to respiratory acidosis and left-sided pneumonia with septic shock. A follow-up of 6 months post-surgery, reported only 2 patients (3 and 4) with positive clinical outcome (AFB smear conversion from positive to negative) while other 2 patients (patient 1 and 2) showed no clinical improvement with persistent smear positive status. Table 3. summarizes the hospitalization characteristics of each patient.

**Table 1:** Demographic and clinical characteristics of patients.

	Patients^a^				
Characteristics	1	2	3	4	5
**Age (years)/Sex**	21/F	26/M	33/F	49/F	25/F
**Median (Interquartile range)**	26 (25–33)				
**History**	Family history of pulmonary TB; patient has past history of extrapulmonary TB at the age of 12 yrs.	None	Family history of pulmonary TB; patient has past history of pulmonary TB at the age of 16 yrs.	None	Past history of pulmonary TB at the age of 17 yrs.
**Number of treatment regimens before surgery**	7	7	4	8	5
**Median (Interquartile Range)**	7 (5–7)				
**Adherence to treatment** b	Irregular medication^c^	Regular medication^d^	Irregular medication	Regular medication	Regular medication
**Previous treatment with a quinolone and injectable agent (other than first-line agents and Streptomycin)** e	Lfx	Mfx, Spafloxacin, Lfx, Km	Ofx, Sparfloxacin, Lfx, Cm	Ofx, Km	Past medical records lost by the patient
**Treatment with other antibiotics** f	Cs, Eto, PAS	Eto, PAS	Eto, Cs, Clr	Eto, PAS, Lzd	Past medical records lost by the patient
**Other complaints**	Fever, cough, breathlessness, vomiting, decrease in appetite	Fever, cough	Cough, increase expectoration during morning hours	Cough, Diabetis	Disturbed sleep, breathlessness
**Risk factors for multiple MTB infection**	Irregular Medication; family history; patient past history; treatment failure	Treatment Failure^g^	Irregular Medication; family history; patient past history; treatment failure	Patient had completed full course of anti-tubercular treatment; has twice reported negative sputum cultures but after 3 months has documented relapse of the disease without default in treatment.	Past history of TB; this episode was considered as a relapse since the patient developed the disease within a few months after completing the treatment regimen.

a All patients were HIV negative.

b All patients received treatment from private medical practitioners. All patients (except patient 2) on diagnosis were initiated on category I treatment regimen as per the guidelines of the National TB programme but with no improvement in condition even after 1 year, they were started on second- and third- line anti-tubercular drugs.

c Irregular Medication, treatment interrupted for more than a month for reasons due to patient.

d Regular Medication, patient took regular prescribed medication as per the instructions of the private medical practitioner.

e First-line agents, Isoniazid, Rifampin, Ethambutol, Pyrazinamide; Lfx, Levofloxacin; Mfx, Moxifloxacin; Km, Kanamycin; Ofx, Ofloxacin; Cm, Capreomycin;

f Cs, Cycloserine; Eto, Ethionamide; PAS, para-aminosalicylic acid; Clr, Clarithromycin; Lzd, Linezolid.

g Treatment Failure, culture or AFB smear microscopy remaining positive or turning positive even after 6 months of treatment.

**Table 2:** Treatment history of each patient pre- and post- pulmonary resection surgery.

			Baseline phenotypic DST report^a^		Phenotypic DST report prior to surgery^b^			
Patient Nos.	Year in which TB was diagnosed	Empirical treatment initiated on diagnosis	Sensitive	Resistant	Sensitive	Resistant	Medication prior to surgery^c^	Medication post surgery^d^
1	2006	H, R, E, Z	Km, PAS, Mfx, Am, Cfz, Cm	S, H, R, E, Z, Eto, Ofx	PAS, Cfz	S, H, R, E, Z, Km, Eto, Ofx, Mfx, Am, Cfz, Cm	S, Eto, PAS, Lfx, Cs	Km, Eto, Mfx, Cs
2	2006	R, E, Z, Km, Eto, Mfx	E, Km, Eto, PAS, Am, Cfz, Cm	S, H, R, Z, Ofx, Mfx	E, Cfz	S, H, R, Z, Km, Eto, PAS, Ofx, Mfx, Am, Cm	H, R, Z, E, PAS, Sparfloxocin	Eto, Am, Cfz, Lzd, Cs
3	2005	R, Z, Eto, Ofx, Lfx, Cs, Clr	S, E, Km, Eto, PAS, Mfx, Am, Cfz, Cm	H, R, Z, Ofx, Cs, Clr	S, Km, Am, Cfz, Cm	H, R, E, Z, Eto, PAS, Ofx, Mfx	Eto, Am, Lfx, Cs, Clr	Eto, Cm, Cs, Clr
4	2006	S, H, R, E, Z	S, R, Em, Km, Eto, PAS, Mfx, Am, Cfz, Cm	H, Ofx	Eto, PAS, Cfz, Cm	S, H, R, E, Z, Km, Ofx, Mfx, Am	H, R, Km, Lzd	H, Km, Lfx, Lzd
5	2006	S, H, R, E, Z	Past medical records lost by the patient		Km, Am, Cfz, Cm	S, H, R, E, Z, Eto, PAS, Ofx, Mfx, Am	H, PAS, Lfx, Cs	Am, Cs, Lzd, Clr

S, Streptomycin; H, Isoniazid; R, Rifampicin; E, Ethambutol; Z, Pyrazinamide; Km, Kanamycin; Eto, Ethionamide; PAS, para-aminosalicylic acid; Ofx, Ofloxacin; Mfx, Moxifloxacin; Am, Amikacin; Cfz, Clofaziamine; Cm, Capreomycin; Lfx, Levofloxacin; Cs, Cycloserine; Clr, Clarithromycin; Lzd, Linezolid.

a Baseline phenotypic DST report, the initial phenotypic DST report provided by the patient during counseling. This test was done at another laboratory with no information about its accreditation.

b This test was performed on the patients sputum specimen, 3–6 months prior to surgery at the ‘Revised National Tuberculosis Control Programme’ accredited Mycobacteriology laboratory at our hospital. The test provided drug susceptibility pattern to 13 drugs: S, H, R, E, Z, Km, Eto, PAS, Ofx, Mfx, Am, Cfz and Cm.

c This medication was prescribed by a private medical practitioner who referred the patient to our hospital for further treatment.

d This medication was prescribed by the consultant chest physician at our hospital.

**Table 3:** Hospitalization characteristics and outcomes of individual patients undergoing pulmonary resection surgery.

	Patients				
Characteristics	1	2	3	4	5^a^
**No. of days of hospitalization during the time of surgery**	17	15	14	13	6
**Median (Interquartile Range)**	14 (13–15)				
**Type of surgery (Affected lung)**	Pneumectomy (Left)	Pneumectomy (Left)	Pneumectomy (Left)	Lobectomy (Lower Left)	Partial pneumectomy (right)
**No. of months from treatment initiation to surgery**	42	36	54	36	36
**Median (Interquartile Range)**	36 (36–42)				
**Reason to undergo surgery**	Persistent smear positive status.	Persistent smear positive status.	Persistent smear positive status.	Avoid complications arising due to hemoptysis.	Continuous discharge of pus from the right lung.
**Outcome** b	No clinical improvement, with persistent smear positive status	No clinical improvement, with persistent smear positive status and spread on infection in the right lung	Positive clinical outcome - positive smear status conversion to smear negative	Positive clinical outcome - positive smear status conversion to smear negative	Expired.

a Patient 5, underwent a partial right pneumectomy 1½ years back in another private hospital, and expired with 72 hours post-surgery due to respiratory acidosis and left-sided pneumonia with septic shock.

b Outcome, is based on the follow-up 6-month post-surgery.

### MGIT TB culture, phenotypic drug susceptibility testing and genetic sequence analysis

All cavities (3 cavities per patient) identified within the resected lung of each patient were excised and were found to be positive on AFB smear examination and by MGIT TB culture with an average time to positivity of 20 days. On the basis of their phenotypic DST results, patients 1, 2, 4 and patients 3, 5 were reported as XDR-TB and MDR-TB cases respectively. Patients with extensive drug resistance had received more number of treatment regimens before undergoing surgery than those patients with multi-drug resistance (mean number of regimens ± Standard Deviation, 6.33±0.57 vs 4±0) (Table 1). Three cavity isolates from each of the 3 patients (patients 1, 3, 4) showed identical drug susceptibility profiles. Patient 2 showed variable drug susceptibility profile for 6 drugs (Km, Am, Cm, Ethambutol [E], para-aminosalicylic acid [PAS], Pyrazinamide [Z]) between its three cavities, while patient 5 showed variable drug susceptibility profile for only 1 drug (PAS). In-house RLBH assay confirmed the variable drug susceptible profile for aminoglycosides (Km, Am, Cm) while repeat phenotypic DST confirmed the variable drug susceptibility pattern for E, PAS and Z. Detailed phenotypic drug susceptibility profile and genetic sequence analysis of all 5 patients has been shown in Table 4.

**Table 4:** Phenotypic drug susceptibility profile and genetic sequence analysis of all cavities of each patient.

Patient Nos.	Cavity Nos. (Lobe)^a^	Phenotypic Drug Susceptibility profile^b^		Genetic sequence analysis: mutation observed (codon number)^c^			
		Sensitive	Resistant	rpoB gene	katG gene	gyrA gene	rrs gene
1	1 (LUL), 2 (LUL), 3 (LUL)	PAS, Cfz	S, H, R, E, Km, Eto, Ofx, Mfx, Am, Cm, Z	C to T (531)	G to C (315)	A to G (94)	A to G (1401)
2	1 (LUL)	Cfz, Z	S, H, R, E, K, Eto, PAS, Ofx, Mfx, Am, Cm	C to T (531)	G to C (315)	A to G (94)	A to G (1401)
	2 (LUL)	E, Km, Am, Cm, Z, Cfz	S, H, R, Eto, PAS, Ofx, Mfx	C to T (531)	G to C (315)	A to G (94)	wt^d^
	3 (LLL)	E, PAS, Cfz	S, H, R, K, Eto, Ofx, Mfx, Am, Cm, Z	C to T (531)	G to C (315)	A to G (94)	A to G (1401)
3	1 (LUL), 2 (LLL), 3 (LLL)	S, Km, Am, Cfz, Cm	H, R, E, Eto, PAS, Ofx, Mfx, Z	C to T (531)	G to C (315)	A to G (94)	Wt
4	1 (LUL), 2 (LUL), 3 (LUL)	Cfz	S, H, R, E, Km, Eto, PAS, Ofx, Mfx, Am, Cm, Z	C to T (531)	G to C (315)	A to G (94)	A to G (1401)
5	1 (RUL)	Km, PAS, Am, Cfz, Cm	S, H, R, E, Eto, Ofx, Mfx, Z	C to T (531)	G to C (315)	A to G (94)	wt
	2 (RUL)	Km, Am, Cfz, Cm	S, H, R, E, Eto, PAS, Ofx, Mfx, Am, Z	C to T (531)	G to C (315)	A to G (94)	wt
	3 (RLL)	Km, Am, Cfz, Cm	S, H, R, E, Eto, PAS, Ofx, Mfx, Am, Z	C to T (531)	G to C (315)	A to G (94)	wt

a LUL, Left upper lobe; LLL, left lower lobe, RUL, right upper lobe; RLL, right lower lobe.

b S: Streptomycin, H:Isoniazid, R:Rifampicin, E:Ethambutol, Km:Kanamycin, Eto:Ethionamide, PAS:para-aminosalicylic acid, Ofx:Ofloxocin, Mfx:Moxifloxacin, Am:Amikacin, Cfz:Clofaziamine, Cm:Capreomycin, Z:Pyrazinamide.

c Cavity isolates showing variable drug susceptibility profiles were analyzed by in-house RLBH (except for Ethambutol, PAS, Pyrazinamide, wherein phenotypic DST were repeated to confirm the variable drug susceptibility profile).

d wt – wild type.

### Genotyping

**Figure 3:**
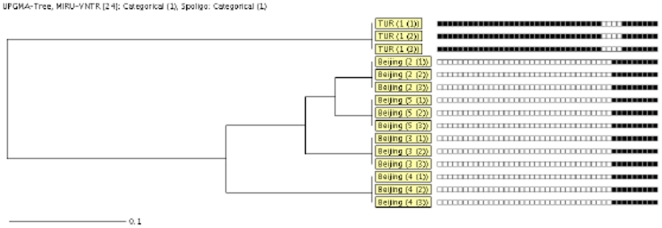
UPGMA tree showing the comparison of the cavity isolates from 5 patients based on the combined similarities of both the 24-loci MIRU-VNTR and spoligotype results determined by using the categorical coefficient. Clustered isolates are labeled with a bar. From left to right are shown: box consisting of the MTB lineage and patient number (cavity no.), and spoligotype pattern.

Since all 5 patients possessed risk factors (Table 1) favoring the presence of multiple MTB infection, both spoligotyping and 24-loci MIRU-VNTR were performed on all cavity isolates of each patient to confirm its presence. But both showed the presence of a clonal population of MTB strain in all cavities of each patient suffering from chronic progressive TB. None of the 24-loci showed the presence of more than one allele at a single locus, also ruling out the probability of presence of sub-clonal population of the infecting strain. Four patients (patients 1, 2, 4, 5) were found to be infected with a strain belonging to the Beijing family while only one patient (patient 3) was infected with a strain of T1 family. Fig. 3. shows a Unweighted Pair Group Method with Arithematic Mean (UPGMA) based phylogenetic tree showing comparison of all cavity isolates from 5 patients based on combined similarities of both the 24-loci MIRU-VNTR and spoligotyping results.

## Discussion

Our findings are similar to that of Kaplan *et al.* [9] that a clonal population of *M. tuberculosis* had localized within the lung forming multiple cavities, causing tissue necrosis and leading to destruction of the lung.

Our study reports that, 2/5 patients (all on ATT from the past 36 months) had different drug susceptibility profile (for E, Km, PAS, Am, Cm, Z) between strains from different cavities of a single patient i.e. some of them were sensitive for a particular drug while others were resistant against that same drug. The resistance profiles of these strains were confirmed on the basis of presence of mutations in the genes responsible for resistance to R, H, Ofx, Mfx, Km, Am and Cm. In-house RLBH revealed the presence of uniform genetic mutations within all cavities for patients reporting resistance to rifampicin (C531T in rpoB gene), isoniazid (G315C in katG gene), fluoroquinolones (A94G in gyrA gene) and aminoglycosides (A1401G in rrs gene). Variable drug susceptibility profiles for Ethambutol, PAS and Pyrazinamide were confirmed by repeating the phenotypic DST rather than on the genetic basis due to the following reasons: 1) genetic analysis for determining Ethambutol resistance targeting emb306 mutation is debatable since its presence has been reported even in sensitive strains and hence was not tested in this study [10], [11], 2) a recent study by Feuerriegel *et al.* [12] and Mathys *et al.* [13] have demonstrated that the much hyped Thr202Ala polymorphism in thyA and mutations in other genes like folP1, folP2, thyX and dfrA are not valid makers for PAS resistance and that other mechanisms are responsible for resistance to PAS, and 3) similarly, genetic analysis for determining PZA resistance is controversial since many PZA resistant isolates have shown to have a wild-type sequence, suggesting that phenotypic DST is essential for determination of PZA resistance [14]. Due to these inconsistent and unpredictable results, phenotypic DST was preferred over genetic sequence analysis to confirm the variable drug susceptibility profile for Ethambutol, PAS and Pyrazinamide.

The variable drug susceptibility profile (heteroresistance) reported between different cavity isolates of the same patient could either be due to multiple MTB strain infection or drug resistance, a result of non-adherence to-/inadequate- treatment regimen. To better understand the basis of such heteroresistance, all cavities isolates were genotyped for better identification; spoligotyping identified strains from 12 cavities of 4 patients belonging to the Beijing family and 3 cavities from a single patient to the T1 family. These findings were confirmed by MIRU-VNTR with absence of double alleles at all 24-loci for all isolates indicating presence of clonal population of MTB strain unlike other studies wherein multiple infection and/or sub-clonal population have been reported in clinical samples [4], [7]. The presence of a clonal population and variable drug resistance profile, indicates that resistance against these drugs had been acquired recently and independently at different focal sites in the lung. Subsequently with time, this drug-resistant strain could overcome the host-immune response and the effects of anti-tubercular drugs to replicate and outgrow the sensitive strain. In such cases, treatment regime should target both drug sensitive- and resistant- organisms to completely act on and kill the drug sensitive organisms. For example, improved outcomes for patients with MDR-TB who were receiving therapy involving Isoniazid together with the other drugs has been reported [15].

This study also documents the use of inadequate treatment regimen to treat M/XDR-TB prior to surgery (table 2). Treatment regimens relied heavily on first-line drugs (H, R) instead of the recommended regimen consisting of a three second-line drugs, 2–3 third line drugs and an injectable to which the isolate is susceptible (if available), in case of extensive drug resistant patients [16]. The extensive drug resistance observed prior to surgery left the surgeon and referral hospital clinicians with restricted ability to adjust drug regimens post surgery. The regimen post surgery included later generation fluoroquinolones, Moxifloxacin or Levofloxacin and/or other third-line agents like Clofaziamine, Clarithromycin, Cycloserine or Linezolid. Although the evidence of positive outcomes of such treatment regimens is limited, we cannot exclude the possibility that they might provide treatment success by increasing the regimens activity or by providing protection to the emergence of resistance to more active agents [17].

The second important finding of the study, in spite of the presence of risk factors (like non-adherence to-/inadequate- treatment, past history and/or contact with an active TB person) indicative of multiple MTB infection, all patients were found to be infected with only a single infecting MTB strain. Four of the 5 patients included in the study were infected by *M. tuberculosis* strain belonging to the Beijing family. Based on the risk factors, we hypothesize that the patient could be infected by multiple MTB strains but over a treatment period of 36 months, the dominant strain of MTB could have outgrown other strains. This dominant strain with time could have undergone genetic evolution by accumulation of mutations conferring drug resistance, thus making the strain resistant to a broad-spectrum of anti-tubercular drugs [18].

Besides the 2 important findings, our study also has several limitations:

1. The small sample size makes it difficult to determine possible risk factors that could lead to chronic progressive disease. Also, all the findings reported need to be confirmed by a study on a larger sample size.
2. The drug susceptibility tests were performed on the culture isolate after *in vitro* growth of MTB strain and not directly on the specimen itself. In presence of multiple MTB infection, the dominant strain may outgrow the other strain. This could lead to an unreliable and inaccurate determination of drug susceptibility pattern, forming the basis for an ineffective treatment regimen.
  This emphasizes the need for development of novel diagnostic tools for determination of drug susceptibility profile directly from clinical specimens.
3. Though 24-loci MIRU-VNTR can detect multiple MTB infections even if one of them is underrepresented at 1% of the total bacterial population, the detection of clonal heterogeneity in clinical isolates is difficult. It requires the analysis of multiple independent colonies unlike this study wherein a single colony isolate was used for genotyping. A recent study by Gardy *et al.* [19], has reported the use of whole genome sequencing in differentiating strains with identical MIRU-VNTR profiles.

In conclusion, the study reports the presence of clonal population of MTB strain, in some cases exhibiting heteroresistance within multiple cavities of a patient. Inadequate-/non-adherence- to treatment regimen, could be an additional risk factor (needs further evaluation) contributing to this form of the disease. These findings also address the need for the development of alternate rapid interventions that will improve treatment outcomes, as well as interrupt the transmission of this extensively drug resistant TB.

## Materials and Methods

### Study setting and Ethical approval

This study was carried out in a private tertiary referral care hospital in Mumbai. This study was approved by the National Health and Education Society, P. D. Hinduja National Hospital and Medical Research Centre. Written consent was also obtained from each patient.

### Patients

Five patients (patients 1–5) unsuccessfully treated for TB underwent pulmonary resection surgery from June 2009 to July 2010 were included in the study. Patient 5 had undergone a partial pneumectomy of the affected lung 1½ years back in another private hospital. Drug susceptibility testing of sputum specimen of each patient was carried out in the Mycobacteriology laboratory of this hospital prior to surgery. Past history and treatment history of each patient was studied.

### MGIT TB culture

On arrival of the untreated lung, all cavities (3 cavities in each of the 5 patients) were identified in the resected lung of each patient and excised using a sterile blade in a biosafety cabinet II. These cavities were processed for mycobacterial culture: processed with *N*-acetyl L-cysteine and sodium hydroxide (NALC–NaOH) [20], cultivated on both solid medium (egg-based Löwenstein– Jensen) [21] and liquid medium (BACTEC MGIT [mycobacteria growth indicator tube] 960 culture; BD Microbiology Systems). Culture positives were confirmed for MTB species by p-nitro benzoic acid assay [21].

### Phenotypic DST and genetic sequence analysis

Phenotypic drug susceptibility testing (DST) for first- [22] and second- line drugs [23] was carried out on all cavity culture isolates of each patient. Isolates from different cavities within a single patient showing variable phenotypic drug susceptibility profile were confirmed either by repeating the phenotypic DST and/or processing the isolates for genetic sequence analysis using in-house Reverse Line Blot Hybridization (RLBH) assay targeting specific gene mutations conferring resistance to first-line drugs like Rifampicin (R) and Isoniazid (H), and second-line drugs like aminoglycosides (Kanamycin [Km], Amikacin [Am], Capreomycin [Cm]) and fluoroquinolones (Ofloxacin [Ofx], Moxifloxacin [Mfx]) [24].

### Genotyping

All cavity culture isolates grown on solid media were genotyped by Spoligotyping (Isogene, Netherlands [as per the manufacturer's instructions]) and 24-loci MIRU-VNTR [25] to confirm the presence or absence of multiple MTB infection.
